# Activity of Heat Shock Genes’ Promoters in Thermally Contrasting Animal Species

**DOI:** 10.1371/journal.pone.0115536

**Published:** 2015-02-20

**Authors:** Lyubov N. Astakhova, Olga G. Zatsepina, Sergei Yu. Funikov, Elena S. Zelentsova, Natalia G. Schostak, Konstantin E. Orishchenko, Michael B. Evgen’ev, David G. Garbuz

**Affiliations:** 1 Engelhardt Institute of Molecular Biology RAS, Vavilov str. 32, Moscow, 119991, Russia; 2 Institute of Cell Biophysics RAS, Pushchino, Moscow region, 142290, Russia; 3 Institute of Cytology and Genetics, The Siberian Branch of RAS, Prospekt Lavrentyeva 10,630090, Novosibirsk, Russia; University of Cincinnati, UNITED STATES

## Abstract

Heat shock gene promoters represent a highly conserved and universal system for the rapid induction of transcription after various stressful stimuli. We chose pairs of mammalian and insect species that significantly differ in their thermoresistance and constitutive levels of Hsp70 to compare *hsp* promoter strength under normal conditions and after heat shock (HS). The first pair includes the *HSPA1* gene promoter of camel (*Camelus dromedarius*) and humans. It was demonstrated that the camel *HSPA1A* and *HSPA1L* promoters function normally *in vitro* in human cell cultures and exceed the strength of orthologous human promoters under basal conditions. We used the same *in vitro* assay for *Drosophila melanogaster* Schneider-2 (S2) cells to compare the activity of the *hsp70* and *hsp83* promoters of the second species pair represented by Diptera, i.e., *Stratiomys singularior* and *D*. *melanogaster*, which dramatically differ in thermoresistance and the pattern of Hsp70 accumulation. Promoter strength was also monitored *in vivo* in *D*. *melanogaster* strains transformed with constructs containing the *S*. *singularior hsp70* ORF driven either by its own promoter or an orthologous promoter from the *D*. *melanogaster hsp70Aa* gene. Analysis revealed low *S*. *singularior hsp70* promoter activity *in vitro* and *in vivo* under basal conditions and after HS in comparison with the endogenous promoter in *D*. *melanogaster* cells, which correlates with the absence of canonical GAGA elements in the promoters of the former species. Indeed, the insertion of GAGA elements into the *S*. *singularior hsp70* regulatory region resulted in a dramatic increase in promoter activity *in vitro* but only modestly enhanced the promoter strength in the larvae of the transformed strains. In contrast with *hsp70* promoters, *hsp83* promoters from both of the studied Diptera species demonstrated high conservation and universality.

## Introduction

It is well known that heat shock proteins (Hsps), particularly members of the Hsp70 family, are involved in thermoprotection at the cellular level, preventing protein aggregation and the refolding or degradation of stress-damaged proteins [1].

During heat shock (HS) and other forms of stress, a battery of HS genes in eukaryotes is induced by an activated heat shock transcription factor (HSF), which binds highly conserved regulatory sequences (heat shock elements, HSEs) located within HS gene promoters. Rapid activation of *hsp* genes leads to drastic changes in the local chromatin structure visualized in the form of large puffs in larval salivary gland chromosomes of various Diptera species including *Drosophila melanogaster* [2]. Puff formation depends not only on HSF binding but also on the presence of other essential factors, including GAGA-binding factor (GAF), which has been shown to disrupt nucleosome structure [3–8]. It was assumed for a long time that *hsp70* promoters are highly conserved in evolution and exhibit similar robust regulatory functions in various heterogeneous systems [9–13]. Along these lines, a previous report provided evidence that the *Drosophila hsp70* promoter is capable of driving heat-inducible transgene expression in the silk moth *Bombyx mori* [13]. Indeed, multiple studies have demonstrated the ability of the *Drosophila hsp70* promoter to drive gene expression in non-drosophila insects and even in non-insect organisms [14–16].

However, in recent years, quantitative studies indicated that the heat-inducible activity of the *Drosophila hsp70* promoter may be low in some non-drosophila insects [12,17].

In our previous studies, we have described the heat shock response at the molecular level in various insects, crustacea, reptiles and mammalian species that drastically differ in the temperature of their ecological habitats.

It is of note that most studies of the possible role of Hsps in thermoresistance were performed using insect species and other poikilothermic organisms whose core temperatures are highly variable. However, in some cases, superficial tissues of mammals vary significantly in temperature when subjected to severe environmental challenges. Thus, in arid desert areas, the skin of camels may exceed 40°C [18]. Previously, we demonstrated that camel lymphocytes constitutively express the HSP73 protein, which can be significantly induced by heat shock. Surprisingly, another member of the HSP70 family (HSP72) is not induced in lymphocytes but can be induced in skin fibroblasts by elevated temperature. It was also shown that the total protein synthesis in camel cells is significantly more resistant to heat than that of human cells [18]. Recently, inducible and constitutive camel *HSP70* genes have been sequenced in our laboratory and compared with orthologous genes from other mammalian species [19]. Herein, we compare the transcriptional activity of the camel and human *HSP70* gene promoters by exploring a luciferase reporter in an *in vitro* assay in two different human cell lines.

Furthermore, we recently studied several species of flies belonging to the Stratiomyidae family that inhabit strikingly different extreme environments at the larval stage including the hot volcanic streams of Kunashir island, the hypersaline lakes of Crimea and the cold lakes of the St. Petersburg area [20]. Characteristically, all of the studied Stratiomyidae species exhibited high thermoresistance at the larval stage that correlated with the high constitutive level of Hsp70 present in their cells under normal physiological conditions, which was only modestly induced by temperature elevation [20]. Thus, *S*. *singularior* larvae dwell in Crimea hypersaline lakes and are capable of surviving extremely high temperatures (up to 47°C) as shown in our previous study [20]. Comparative analysis of the 5’-regulatory regions of the *D*. *melanogaster* and *S*. *singularior hsp70* genes demonstrated that they are highly divergent in contrast to *hsp83* promoters in the same species [21,22].

Therefore, in the second part of our study, we examined the transcription activity of regulatory regions of the *hsp70* and *hsp83* genes of *S*. *singularior* in comparison with those of *D*. melanogaster. The latter species exhibits only trace amounts of Hsp70 under normal conditions and a drastic increase in the Hsp70 level after HS.

In our previous study, we investigated the patterns of Hsp synthesis in all of the abovementioned species under normal conditions and after HS and cloned and sequenced the *Hsp* genes from these species [19–22]. Based on accumulated data, we decided to perform a comparison using the same *in vitro* assay of the transcriptional activity of the *Hsp70* gene promoters from two pairs of species (Diptera and mammals) that drastically differ in thermoresistance and patterns of Hsp70 induction and synthesis under normal physiological conditions.

Furthermore, taking into account the drastic differences detected in the architecture of the *hsp70* promoters in the two Diptera species, it was of interest to check whether the *S*. *singularior hsp70* promoters could effectively drive transcription in *Drosophila* cells not only *in vitro* but also *in vivo*. Taken together, this study demonstrated that the strength of the *S*. *singularior hsp70* promoter is many-fold lower *in vitro* and *in vivo* than that of the endogenous *D*. *melanogaster* promoter, while the *hsp83* promoters in the compared Diptera species exhibited similar strength in the same *in vitro* assay. The observed differences in *hsp70* promoter transcription activity, at least *in vitro*, was apparently due to the absence of functional GAGA elements within the *S*. *singularior hsp70* regulatory region.

## Results

### *In silico* analysis of regulatory regions of the *hsp70* and *hsp83* genes of different origin

The programs MEME and MatInspector (GENOMATIX Software) were used to align the *HSPA1* regulatory sequences of camel with corresponding orthologous human genes. Therefore, major regulatory elements such as TATA boxes, HSEs, Sp1 and NF-Y-binding sites were determined in the promoters of the *HSPA1A*, *HSPA1B* and *HSPA1L* genes, which comprise a cluster linked to the major histocompatibility complex (MHC) locus in all mammalian species studied thus far. In the course of this analysis, we detected a 112 bp insertion in the *hspA1L* gene promoter of humans and other primates (e.g., *Gorilla gorilla* and chimpanzee *Pan troglodytes*) [19]. Intriguingly, this primate-specific sequence includes two homeodomain-binding sites. Despite the high homology of *hsp70* regulatory regions observed in vertebrates, particularly mammalian species, there are a certain number of deletions, insertions and substitutions that discriminate the species *hsp70* promoters and may in principle underline different promoter strengths and specificity even within mammalian species (S1 Fig.)

In contrast to the studied mammalian species, the Diptera species used in this study drastically differ in the arrangement of the *hsp70* promoters while exhibiting a similar architecture for hsp83 gene promoters. In our previous study, we generated a lambda library from the genomic DNA of *S*. *singularior* and sequenced five *S*. *singularior hsp70* genes designated S1—S5, which are located in tandem or are in an inverted orientation and comprise the *Hsp70* gene cluster in this species [21]. All *hsp70* genes in *D*. *melanogaster* contain highly homologous promoter regions, with four similarly spaced HSEs localized at the same distance from the TATA box [23]. On the other hand, the orthologous genes in *S*. *singularior* share only comparatively conserved coding regions and the first 50 bp upstream of the transcription start site, which includes a TATA box and the first HSE (see S2 Fig.). In contrast with known *Drosophila* species, in *S*. *singularior hsp70* genes, the regions upstream of the transcription start sites and the first HSE and downstream of the stop codon (50 bp area) do not exhibit significant homology, and 5’-flanking regions contain different number and spacing of HSEs [21] (see also S2 Fig.). In addition to HSEs, the *hsp70* regulatory regions (130 bp upstream the TATA box) in *D*. *melanogaster* and other *Drosophila* species usually contain several GAGA-sites, which are necessary for rapid transcription induction [24]. Functional GAGA sites in *Drosophila* are usually represented by GAGAG or GAGAGAG motifs or contain several GAG trinucleotides separated by spacers with an uneven number of nucleotides (one, three or five) [25]. The search performed for canonical GAF-binding sites using the MatInspector program failed to detect the presence of these sequences in *S*. *singularior hsp70* promoters. In contrast to *Drosophila* species, orthologous *S*. *singularior* promoters contain only single GAG motifs. Furthermore, the TATA box in *S*. *singularior hsp70* promoters is represented by a TATATA sequence in contrast to the TATAAA motif observed in *Drosophila*. The analysis failed to detect any additional known regulatory motifs in common within the promoters of all five *S*. *singularior hsp70* genes. Interestingly, another pair of orthologous *hsp* genes (*i*.*e*., *hsp83*) present in both compared species (*S*. *singularior* and *D*. *melanogaster*) exhibits comparatively high homology and shares a similarly arranged single HSE found at nearly the same distance from the TATA box [22].

### Camel *hsp70* promoters normally function in human cells and exceed the strength of human promoters under steady conditions

**Fig 1:**
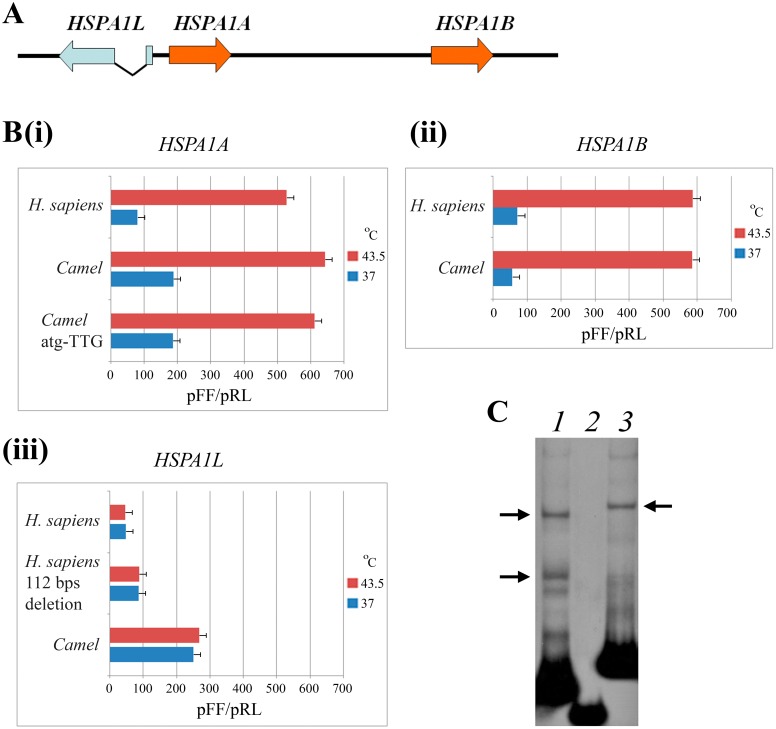
A. General arrangement of the *HSPA1* cluster in mammals. B. The level of transcription activity of individual *hsp70* gene promoters comprising the cluster based on the measurement of transient luciferase luminescence driven by constructs carrying different human and camel *hspA1* promoters. (i)—*HSPA1A*, (ii)—*HSPA1B* and (iii)—*HSPA1L*. The signal levels are shown as the ratio of the intensity of the luminescence of firefly (pFF) and renilla (pRL) luciferase. C. EMSA experiments with human and camel *HSPA1L* promoters fragments: 1—*H*. *sapiens* promoter without 112 bp fragment specific for primates only, 2—isolated *H*. *sapiens* 112 bp fragment, 3—*C*. *dromedarius* promoter. *** and ### p ≤ 0.001, ** p ≤ 0.01, * p ≤ 0.05.

It is known that in mammals, *hsp70* family genes belonging to the *HSPA1* cluster are linked with the MHC class III region and include three genes. Two of these genes (*HSPA1A* and *HSPA1B*) form a tandem repeat and are stress induced, while the third (*HSPA1L*), which is localized in an inverted orientation, is constitutively expressed (Fig. 1A) [19,26].

We developed sixteen constructs where a reporter gene (firefly luciferase) ORF was placed under the control of *HSPA1* group promoters of different origin and architecture (see S3 Fig. for details). Specifically, to investigate the transcriptional activity of inducible *HSPA1A* promoters, we generated five constructs that included different regulatory regions from corresponding human and camel genes. The first two constructs included the camel *HSPA1A* regulatory regions of different lengths (-413…+31 bp and-413…+192 bp in relation to the transcription start site). Analogous constructs for the human *HSPA1A* gene comprised-505…+25 bp and-505…+221 bp. These constructs include two HSEs, a TATA box, and several motifs providing constitutive expression of mammalian *hsp70* genes such as Sp1- and NF-Y-binding sites (S3 Fig.). Because our previous analysis revealed an additional potential start codon (ATG) in the camel *HSPA1A* 5`-UTR [19], in the fifth construct, we substituted it for TTG to examine whether such a mutation would somehow influence gene expression.

Luminescence measurements indicated that under normal physiological conditions in human HEK293 cells (similar results were obtained in human HDF cell culture, data not shown) transformed with the obtained constructs, the activity of the camel *HSPA1A* promoter significantly exceeded (two times) the activity of the orthologous human promoter (Fig. 1Bi). However, after temperature elevation, the expression level of both constructs was similar. The mutation of the additional upstream ATG within the camel *HSPA1A* 5`-UTR did not influence construct expression; hence, this site apparently has no functional significance.

Analysis of the promoter activity of the second inducible gene in the cluster (*HSPA1B*) was performed exploring two constructs of different lengths for the camel gene (-738…+8 bp and-738…+195 bp with respect to the transcription start site) and two analogous constructs for the human gene comprising-733/+35 bp and-733…+206 bp (S4 Fig.). Measurements of promoter strength in the luciferase assay failed to reveal significant differences in the compared constructs either under normal conditions or after heat shock (Fig. 1Bii). The described constructs contained two HSEs, and in general, heat shock led to a 5–6-fold increase in the expression of all of the *HSPA1A* and *HSPA1B* constructs regardless of the species of origin (the results of all construct activity measurements are summarized in Fig. 1Bi, Bii and Biii).

At the next stage, we investigated the strength of the camel and human promoters isolated from the constitutively expressed *HSPA1L* gene arranged in an inverted orientation in relation to the inducible members in the cluster (Fig. 1A). It was interesting to examine whether the HSEs located in the *HSPA1A* promoter somehow influenced the expression of the adjacent *HSPA1L* gene after HS. It has been previously shown that HSEs may in principle enhance transcription bidirectionally [23,27]. Therefore, we developed a series of seven constructs including four of that had the entire regulatory region between *HSPA1A* and *HSPA1L* genes differing by the length of the 5`-UTR (S3 Fig.). In the two other constructs, HSEs responsible for transcription induction after HS were experimentally deleted (S3 Fig.). Finally, in the last construct obtained from the human *HSPA1L* gene, a 112 bp sequence detected only in primates was deleted to monitor its possible impact on promoter activity (-142…-254 bp in human, see S1 Fig. and S3 Fig.). Luminescence measurements results demonstrated that the *HSPA1L* promoters of both species are not induced by HS in HEK293 and HDF cells, while the camel promoter exhibited at least 5-fold higher activity under normal conditions and after HS when compared with the orthologous human promoter (Fig. 1Biii). Interestingly, deletion of the 112 bp sequence in the human promoter led to a statistically significant increase (two-fold) in construct expression compared with the intact promoter (Fig. 1Biii).

To determine whether any transcription factors bind this sequence (112 bp) specific for the human promoter, EMSA experiments were performed using various labeled fragments of the *HSPA1L* regulatory region of both species with protein extract from HEK293 cells. Such analysis failed to demonstrate any binding of the human 112 bp sequence with regulatory proteins in HEK293 cells (Fig. 1C). We speculate that the observed enhanced luminescence after experimental deletion may be explained by the fact that a second Sp1-binding site becomes located closer to the transcription start after deletion of the 112 bp sequence (S3 Fig.).

Notably, partial deletion of the 5`-UTR decreases luminescence in the case of the human *HSPA1A* and *HSPA1B* and camel *HSPA1B* genes but has apparently no effect on camel *HSPA1A* genes and the constitutively expressed *HSPA1L* copies in both species (S3 Fig. and S4 Fig.).

Fig. 1B summarizes the data for the promoter strengths of the human and camel *hspA1* gene cluster under normal conditions and after HS and demonstrates similar transcriptional activity for camel promoters in heterologous human cell systems, which suggest, among other explanations, a high level of conservatism and universality for *HSPA1* group promoters in distant mammalian species.

### *S*. *singularior hsp70* promoters exhibit low activity in contrast with those of *hsp83* in *D*. *melanogaster* cell culture

Previously, we have shown that all sequenced *S*. *singularior hsp70* genes differ in their regulatory regions in contrast to the six known *D*. *melanogaster hsp70* genes, which have nearly identical promoter sequences [21]. To reveal the role of the observed characteristic differences in the structure of the *D*. *melanogaster* and *S*. *singularior hsp70* regulatory regions, we investigated the ability of these highly divergent species-specific *hsp70* promoters to drive constitutive and heat-induced expression of the luciferase gene in Schneider-2 (S2) *D*. *melanogaster* cell culture.

To reach this goal, we developed constructs with the luciferase ORF under the control of *hsp70* promoters with different architecture (S5 Fig.). Fortunately, the 3′-UTRs of all five *hsp70* genes from *S*. *singularior* are unique, and in our previous study, we performed 3′-RACE-analysis and demonstrated that all five of the genes are transcribed under heat shock conditions [21]. In the construct designated *hsp70S3*, the luciferase ORF was placed under the control of the *S*. *singularior hsp70S3* gene promoter. We also used a promoter from another *S*. *singularior hsp70* gene (*hsp70S4*) to examine whether the low activity observed in *D*. *melanogaster* cells was due to a specific structure in the *hsp70S3* gene or if it represented a characteristic of different *Stratiomys hsp70* genes. Our analysis indicates that promoters of *hsp70S3* and *hsp70S4* genes exhibit similar “strengths” when tested in the luciferase reporter system. The reporter construct with a promoter from the *D*. *melanogaster hsp70Aa* gene served as a positive control in these experiments. With the exception of the upstream regulatory 5’-region, all constructs contained the 5’-UTRs necessary for normal translation under HS conditions (S5 Fig.). Previously, we demonstrated that the *S*. *singularior* and *D*. *melanogaster Hsp70* mRNAs have similar stability [19].

**Fig 2:**
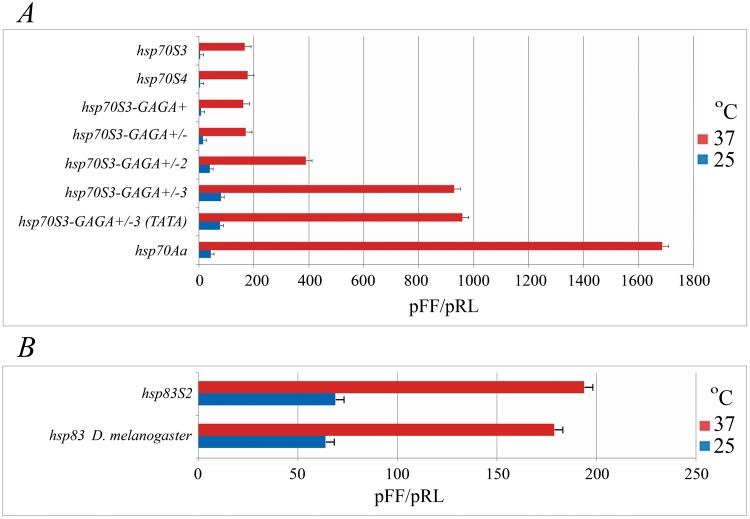
A. Various *hsp70* promoter strengths based on the luciferase luminescence levels in S2 cells and the effects of experimental GAGA element insertion into the *hsp70S3* promoter. B. The level of *S*. *singularior* and *D*. *melanogaster hsp83* promoter activity in *D*. *melanogaster* S2 cells. *** p ≤ 0.001, ## p ≤ 0.01.

Our experiments monitoring *in vitro* transcription levels (luminescence) demonstrated that the *D*. *melanogaster hsp70Aa* promoter has at least ten-fold higher activity in S2 cells, both under steady-state conditions and after HS (37°C) compared with the *hsp70S3* and *hsp70S4* promoters (Fig. 2) in the luciferase expression system. Because we detected drastic species-specific differences with regards to the *hsp70* promoter activity in S2-based transient transcription assays, it was of significant interest to determine whether other *hsp* genes in these species follow the same pattern (i.e., much lower strength for *S*. *singularior* promoters). Therefore, we generated two constructs containing either the 318 bp sequence of the *S*. *singularior hsp83S2* promoter (-155…+163 relative to the transcription start site) or the 297 bp sequence of the *D*. *melanogaster hsp83* promoter (-147…+150). The constructs are depicted in S5 Fig., and the primers used are shown in S1 Table. Surprisingly, in contrast with the results of the *hsp70* promoter studies, similar transfection experiments with subsequent luminescence measurements demonstrated that the *hsp83* promoters of both species exhibit similar strength under normal conditions (25°C) and after HS (37°C) (Fig. 2). The obtained results coupled with recent data on the structure of *Hsp83* promoters [22, 23] indicate that the *hsp83* promoters of the compared distant Diptera species apparently preserve a high level of conservation; hence, they are capable of driving the transcription of the reporter gene with nearly equal efficacy in cells of the same host species (*D*. *melanogaster*).

### *S*. *singularior hsp70* promoters are capable of binding *Drosophila* HSF but not GAF in EMSA assay

It was interesting to reveal the molecular mechanisms underlying the dramatic differences in transcription activity between the *D*. *melanogaster* and *S*. *singularior hsp70* promoters observed *in vitro* in *Drosophila* cell culture. First, it was necessary to determine whether the promoter of the *hsp70S3* gene is capable of binding *Drosophila* HSF. This promoter contains three HSEs at positions similar to those in the *D*. *melanogaster hsp70* genes but drastically different from the *D*. *melanogaster hsp70* promoters in the context of surrounding sequences. Besides, *hsp70S3* gene promoter has a different transcription initiator and a few substitutions in the HSEs (S2 Fig.) [21].

**Fig 3:**
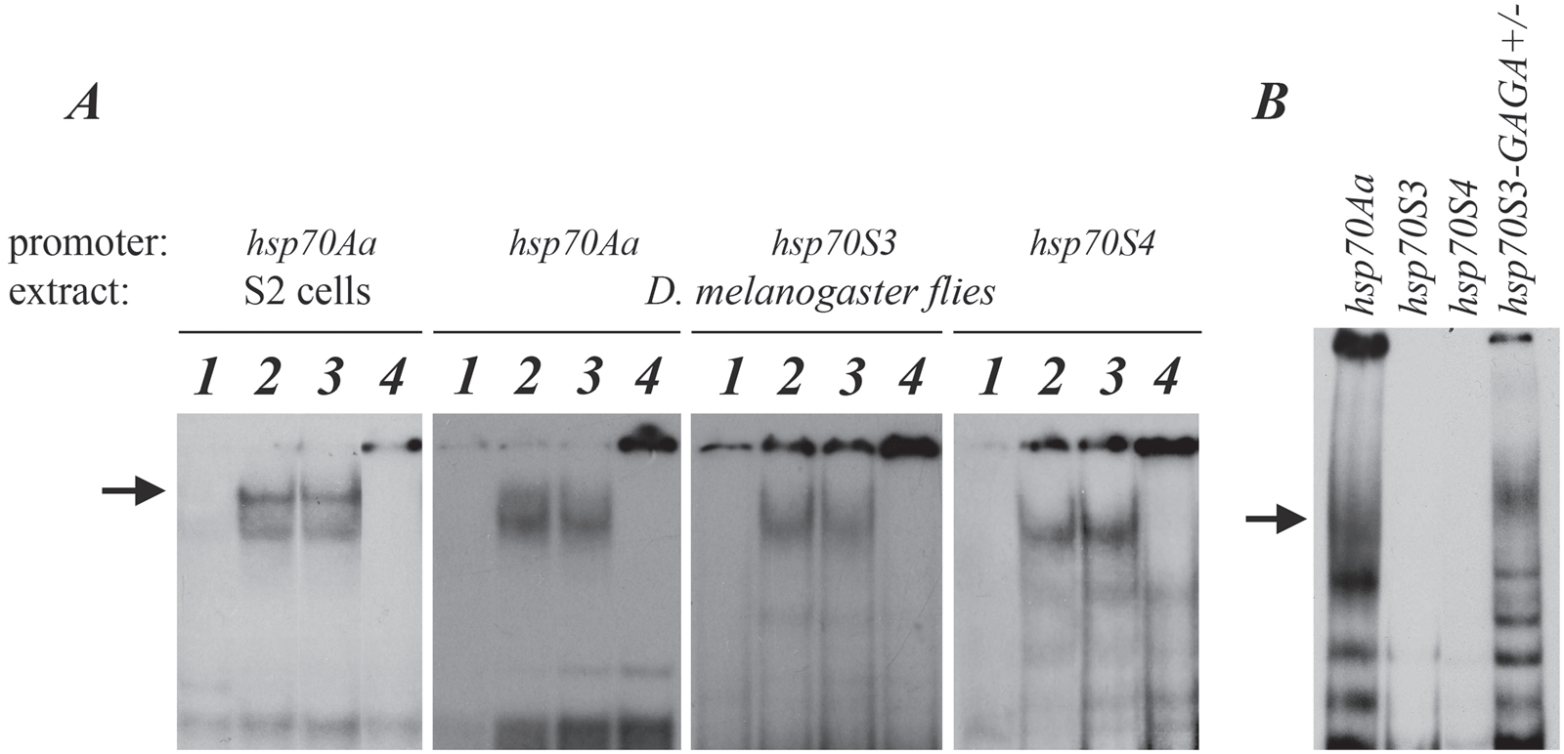
A. EMSA experiments with protein extracts from S2 cells and adult *D*. *melanogaster* flies exploring labeled fragments of the *D*. *melanogaster hsp70Aa* and *S*. *singularior hsp70S3* and *S4* genes. 1—Control (25°C), 2—heat shock (37°C), 3—heat shock + preimmune serum, 4—super shift with anti-HSF. The arrow indicates the position of the HSF-HSE complex. B. Recombinant *D*. *melanogaster* GAF protein effectively binds to the *D*. *melanogaster hsp70* and *S*. *singularior hsp70S3* promoters with the experimental insertion of three GAGA elements (lanes 1 and 4) but not with the wild-type *hsp70S3* and *hsp70S4* promoters (lanes 2 and 3). The arrow indicates the position of the GAF-GAGA complexes.

EMSA experiments with protein extracts from heat-shocked *D*. *melanogaster* flies and S2 cells are depicted in Fig. 3A. It is evident that sequences-94 to-39 bp relative to the transcription start sites in the *S*. *singularior hsp70S3* and *hsp70S4* promoters are capable of binding the *D*. *melanogaster* HSF factor, similarly to the orthologous region taken from the *D*. *melanogaster hsp70* regulatory zone used as a positive control (Fig. 3A). Furthermore, the addition of anti-HSF antibodies prevents the HSF-HSE complex from migrating into the gel (supershift), verifying the binding specificity (Fig. 3A). Therefore, we conclude that the detected differences in promoter strengths apparently do not depend on the different abilities of the *D*. *melanogaster* and *S*. *singularior hsp70* promoters to bind the *Drosophila* HSF.

GAGA-binding factor (GAF) is another candidate factor that may be responsible for the observed differences. GAGA elements are essential sequences found in *D*. *melanogaster hsp70* promoters and a few other HS genes of various *Drosophila* species [24,28,29]. While GAF is necessary for the induction of *hsp70* genes in *Drosophila* [24], canonical GAGA motifs were not detected in orthologous genes in *S*. *singularior* (S2 Fig.). On the other hand, we detected a few GAG or CTC sequences within the *S*. *singularior hsp70* regulatory regions, and there was evidence that under certain conditions, such triplets may also bind GAF with low efficiency [30].

To determine whether *Drosophila* GAF is capable of binding *S*. *singularior* promoters, we performed EMSA experiments using purified recombinant *D*. *melanogaster* GAF. In this series of experiments, we used PCR fragments from the promoters of the *hsp70S3* and *hsp70S4* genes with orthologous *D*. *melanogaster* sequences as a positive control. The results demonstrated that the promoters of the *hsp70S3* and *hsp70S4* genes were not capable of binding *D*. *melanogaster* GAF, while, as expected, normal binding was observed for endogenous *D*. *melanogaster* promoter sequences containing functional GAGA elements and an *hsp70S3* promoter with an experimentally inserted canonical GAGA site (Fig. 3B).

We hypothesized that the absence of functional GAGA elements in the *S*. *singularior hsp70* promoter may contribute to the observed dramatic differences in the transcription of the *hsp70* promoter-fused constructs under control conditions and after HS in *D*. *melanogaster* S2 cells.

### Experimental insertion of GAGA elements into the *S*. *singularior hsp70* promoter enhances its efficacy in the luciferase assay *in vitro*

To determine whether the presence of functional GAGA elements is the cause of drastic differences in the strength of the *Drosophila* and *S*. *singularior hsp70* promoters in *D*. *melanogaster* cell culture after HS, we inserted different numbers of canonical *D*. *melanogaster* GAGA elements into the *hsp70S3* regulatory region (see S5 Fig.) and monitored the transcriptional activity of the resulting promoters using a luciferase assay.

The insertion of a single functional GAGA-element in the plus orientation at position-65 bp from the transcription start site (similar to its localization in the *D*. *melanogaster hsp70* promoters) did not significantly enhance *hsp70S3* promoter activity after HS and only slightly increased promoter activity under control conditions (construct *hsp70S3-GAGA+* in Fig. 2 and S5 Fig.). The insertion of a second GAGA element in the opposite orientation-145 bp from the transcription start site (*hsp70S3-GAGA+/-*) also did not influence the activity of the promoter after HS but resulted in a nearly four-fold increase in transcription under basal conditions. Furthermore, the construct (*hsp70S3-GAGA+/-2*) containing the insertion of two GAGA elements at-65 (“+” orientation) and-120 (opposite orientation) bp exhibited a two-fold increase in transcription after HS and a 10-fold increase under basal conditions in comparison with the original *S*. *singularior hsp70S3* promoter in S2 cells. Therefore, the presence of two GAGA elements in the opposite orientation-55 bp from the transcription site resulted in transcription driven by the modified promoter under basal conditions, which is equal to that of the endogenous *D*. *melanogaster* promoter, and there was a mild increase in activity after HS (Fig. 2). Finally, the insertion of three functional GAGA elements at-65 (“+” orientation), -120 (“-”orientation) and-145 (“-”orientation) led to a 20-fold increase in transcription levels under steady-state conditions and a 5.6-fold increase after HS in comparison with the original *hsp70S3* promoter (Fig. 2, *hsp70S3-GAGA+/-3* construct). In other words, the *hsp70S3* promoter containing the insertion of three functional GAGA elements similar to their localization in the *D*. *melanogaster hsp70* promoter is capable of driving heat-induced expression in *D*. *melanogaster* S-2 cells with only two-fold less activity than the endogenous *D*. *melanogaster* promoter (Fig. 2). Characteristically, the modified *hsp70S3* promoter in the S2 transient expression system significantly exceeded the activity of the wild-type endogenous *D*. *melanogaster* promoter under steady-state conditions. Importantly, the *hsp70S3* promoter sequence containing experimentally inserted GAGA elements bound *D*. *melanogaster* GAF almost as efficiently as the *D*. *melanogaster hsp70* promoter (Fig. 2). It was important to determine whether the pattern of the *hsp70* activities observed *in vitro* in *Drosophila* cells (S2) using artificial plasmid-based constructs would coincide with the promoter activities determined *in vivo* in transgenic strains, which have normal nucleosome structure and complex chromatin architecture.

### The difference in the TATA box structure of the S. singularior *hsp70* promoter has no functional significance

Notably, the regulatory regions of all cloned *S*. *singularior hsp70* genes are different from the *D*. *melanogaster* orthologs in the structure of the TATA box (TATATA in *S*. *singularior* vs TATAAA in *D*. *melanogaster*). To examine the possible functional significance of the observed differences in TATA boxes between the two species, a construct was generated in which the last T *S*. *singularior hsp70* promoter was substituted by an A, making the motif identical to that in *D*. *melanogaster*. This construct also includes the insertion of three GAGA motifs and was designated *hsp70S3-GAGA+/-3 (TATA)* (S5 Fig.). Substitution of the *hsp70S3* TATA box for that of *D*. *melanogaster* did not lead to increased activity for the *hsp70S3* promoter containing three inserted GAGA elements (Fig. 2); hence, the observed difference in the TATA box structure most likely has no functional importance in this system.

### *S*. *singularior hsp70* promoter exhibits low activity in *D*. *melanogaster* transformed strains

Because the active state of chromatin and the nucleosome structure represent the most important factors providing the high activity of *hsp70* gene promoters, it was important to determine whether the patterns of *hsp70* promoter activity described in the above *in vitro* expression experiments exploring plasmid-based constructs resemble promoter strength *in vivo*.

**Fig 4:**
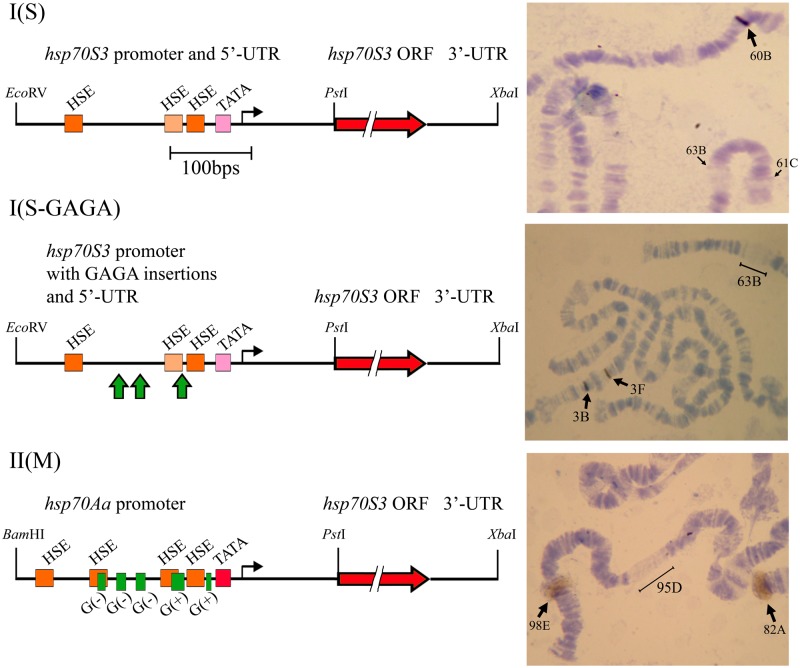
Left panel: General arrangement of constructs used in the transformation experiments. The thick green arrows in I(S-GAGA) indicate the position of the inserted GAGA elements. HSE, GAGA elements and TATA boxes are indicated by square boxes of different color. Right panel: *in situ* hybridization of heat-shocked salivary gland chromosomes with the *white* gene fragment included in the constructs. The sites of the inserts are shown by arrows with puffs formed only in the strains (the bottom panel) containing constructs with *D*. *melanogaster hsp70* promoters. In all panels, the heat shock puffs formed in the locations of major *D*. *melanogaster hsp* genes (i.e., 63B, 61C and 95D) that represent an internal control are indicated. 3B is the *white* locus that hybridizes with the labeled probe and represents an internal control for hybridization efficiency. In each strain, at least ten larvae were used for puff detection after HS.

To reach this goal, we compared the activity of endogenous *hsp70* promoters with that of *S*. *singularior* in several transgenic strains of *D*. *melanogaster* obtained using *P* element mediated transformation as described in the Materials and Methods. Importantly, in all transformation experiments, we used strains with all copies of *hsp70* genes deleted [31]. In the development of transgenic strains, we used the construct I(S) containing the ORF and 5’-UTR of the *S*. *singularior hsp70S3* gene driven by its own intact promoter, the construct I(S-GAGA), which is similar to I(S) but with three GAGA insertions, designated the *hsp70*S3*-GAGA+/-3* construct, and finally the construct II(M) in which the *S*. *singularior hsp70* promoter and 5’-UTR were replaced by orthologous *D*. *melanogaster* sequences linked to the *S*. *singularior* ORF (Fig. 4). We have chosen the *hsp70S3* gene for these comparative analyses because its promoter exhibits maximal deviation from the consensus structure of the *D*. *melanogaster hsp70* promoter [21].

The *hsp70S3* promoter includes three HSEs similarly spaced as in the *D*. *melanogaster hsp70* regulatory region that contains four HSEs (S2 Fig.). However, in contrast with all *D*. *melanogaster hsp70* promoters, the *hsp70S3* promoter and other *S*. *singularior hsp70* promoters do not contain canonical GAGA elements in the plus (GAGAGAG) or minus (CTCTCTC) orientation but includes only a few scattered triplets of the GAG or CTC type.

We obtained three transgenic strains with the *S*. *singularior hsp70S3* ORF under its own promoter (I(S) transgene), designated Sa, Sb and Sc; two strains with the I(S-GAGA) transgene, designated 1G and 3G; and three strains with a construct in which the *S*. *singularior* promoter was replaced by a *D*. *melanogaster* variant (II(M) construct), designated Ma, Mb and Mc. The results of *in situ* hybridization with larval salivary gland polytene chromosomes from all three types of transgenic strains are depicted in Fig. 4 and Table 1. Notably, the obtained transgenic strains usually harbored one or two insertions. 5`-RACE analysis confirmed normal structure for the 5’-ends of mRNAs by sequencing resulting PCR fragments transcribed from the I(S), I(S-GAGA) and II(M) constructs in all obtained transgenic strains (data not shown). It has been demonstrated in many studies that after temperature elevation in the strains transformed by constructs under heat shock promoters typical heat shock puffs are formed not only at sites of well-known heat shock loci but in sites of the inserted constructs as well [7].

**Table 1:** List of transgenic strains used in the investigation.

Transgenic strain symbol	Number of insertions	Localization in the genome
Sa	2	60B^π^ (2R ch.) and 12DE (X ch.).
Sb	1	61A (3L ch.)
Sc	1	37B (2L ch.)
1G	1	12C (X ch.)
3G	1	3F (X ch.)
Ma	2	98E (3R ch.) and 82A (3R ch.).
Mb	1	82A (3R ch.)
Mc	1	98E (3R ch)

^π^–All localizations were performed using Lefevre’s map of *D*. *melanogaster* salivary glands polytene chromosomes [53]. Ch.-chromosome.

Heat shock experiments performed herein demonstrated that while normal prominent puffs are formed in *D*. *melanogaster* polytene chromosomes after HS (37°C) where the II(M) construct with an endogenous promoter was inserted, no puffs were observed at the sites of construct I(S) insertions where the *Hsp70* ORF was under the control of the *S*. *singularior* wild-type promoter or at the sites of I(S-GAGA) transgene insertions (Fig. 4).

**Fig 5:**
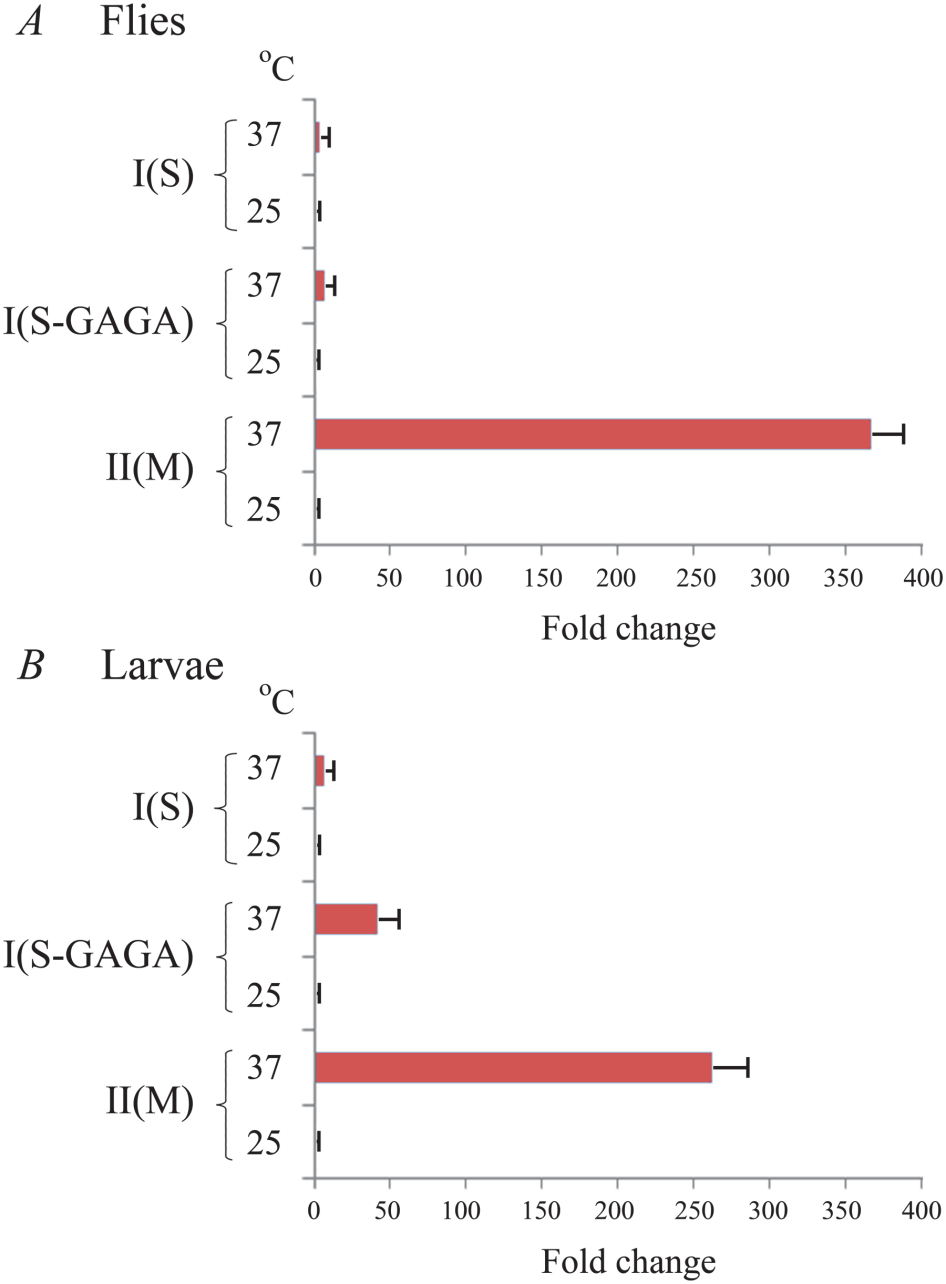
The results of RT-PCR experiments using RNA isolated from adult flies (A) or the third instar larvae (B) of *D*. *melanogaster* strains transformed with constructs containing different *hsp70* promoters. A significant dichotomy in the expression of the I(S-GAGA) construct in adults and larvae is evident, while all other constructs exhibit similar strengths at both stages. *** p ≤ 0.001.

The extremely low heat-inducibility of the *S*. *singularior hsp70* promoter in the *D*. *melanogaster* transgenic strains was further corroborated by Q-RT-PCR experiments. We detected low levels of *hsp70* mRNA under normal conditions and a modest induction of its synthesis in the I(S) transgenic strains after temperature elevation (Fig. 5). Thus, in these strains, HS (37°C for 30 min) induced a slight induction in the *hsp70* mRNA synthesis (3–4-fold). In Ma—Mc strains as expected, we detected several hundred fold induction after HS. Characteristically, similar patterns of induction in both types of transformed strains, i.e., I(S) and II(M), were detected in the larvae and in imagoes (Fig. 5).

In contrast, while the strains transformed with constructs containing the *S*. *singularior* promoter with inserted GAGA motifs did not exhibit significant induction after HS at the imago stage, forty-fold induction was observed at the larval stage. However, the level of induction in the larvae of both strains transformed with the I(S-GAGA) constructs remained 6–7 times lower than in M-strains containing constructs with the endogenous *D*. *melanogaster hsp70* promoter (Fig. 5B). Therefore, while in the case of *in vitro* transient expression, the insertion of three consensus GAGA elements into the *S*. *singularior hsp70* promoter strongly enhanced its activity and rendered it comparable to the orthologous *D*. *melanogaster* promoter (Fig. 2) in *in vivo* experiments exploring transformed strains, a similar insertion resulted in modest induction observed only in the larvae (Fig. 5). Characteristically, this mild increase in promoter strength was not accompanied by heat-induced puff formation in contrast with the constructs of the II(M) type containing *D*. *melanogaster* endogenous promoter (Fig. 4). Thus, the inclusion of the consensus GAGA elements into the *S*. *singularior hsp70* promoter does not lead to its normal functioning in the *D*. *melanogaster* genome *in vivo* but resulted in a modest increase in the promoter strength after HS observed only in the larvae of the transformed strains.

## Discussion

During the last two decades, we have been involved in a study of the heat shock response at the molecular level in various animal species from contrasting thermal habitats. Our analysis and the results of many other groups demonstrated that species usually living under extreme or rapidly changing thermal conditions are characterized by high constitutive levels of Hsp70 and other Hsps in their cells [32]. Such correlation suggests an important role for Hsps and particularly Hsp70 in whole body adaptation to fluctuating thermal conditions.

We decided to compare the architecture and strength of the promoters of two pairs of distant animal species exhibiting similar differences in thermoresistance and patterns of Hsp70 induction.

The *in vitro* analysis of *hsp70* promoter strengths of the first pair (camel and human) demonstrated that the promoters of both species have similar activity after HS, while the strength of the camel promoters (*HSPA1A* and *HSPA1L* genes) was two-fold and five-fold higher, respectively under normal physiological conditions when tested in human cells. The alignment of regulatory region sequences demonstrated high similarity between the two species and the presence of practically identical sets of presumptive regulatory motifs and protein-binding sites. Furthermore, computer analysis revealed the presence of Sp1 binding sites (GC boxes) in human and camel *HSP70* promoters [19]. The existence of this class of regulatory elements was demonstrated in numerous gene promoters that associate with Pol II prior to transcriptional activation [33–36].

Interestingly, in our studies, a primate-specific 112 bp sequence was described in the promoter of the constitutively expressed *HSPA1L* gene [19]. Deletion of this sequence resulted in a two-fold increase in the *in vitro* human promoter activity under basal conditions. We failed to detect any specific regulatory proteins that bind to this region and may underline the observed differences in promoter strength and speculate that deletion of the 112 bp sequence brought the Sp-1 motif closer to the transcription start site, hence enhancing transcriptional activity.

Taken together, our data on promoter strength accumulated in the *in vitro* assay nicely correlated with our previous results demonstrating a higher level of constitutive *HSP70* gene expression in camel cells, which correlates with their higher resistance in terms of total protein synthesis after HS in comparison with human cells [18].

Another pair of *hsp70* promoters used in this study belongs to two distant Diptera species (*D*. *melanogaster* and *S*. *singularior*). *S*. *singularior* and other studied representatives of the Stratiomyidae family are characterized by exceptionally high thermoresistance and high constitutive levels of Hsp70 in larvae [20]. Therefore, the pattern of Hsp70 protein accumulation in the Stratiomyidae species studied to date drastically differs from the *Drosophila* pattern, where Hsp70 is not usually detectable under non-stress conditions but can be dramatically induced by high temperature and other stress factors [20]. In contrast, Northern hybridization analysis and RT-PCR indicated that although all Stratiomyidae species have high constitutive levels of Hsp70, the level of the corresponding mRNA is barely detectable under control conditions, and it is strongly induced by increased temperature just as in *D*. *melanogaster* cells [20].

The comparison of the orthologous promoter transcriptional activity demonstrated low, barely detectable activity for *S*. *singularior hsp70* promoters when monitored *in vitro* in *D*. *melanogaster* S2 cells after HS and under basal conditions. The comparison of regulatory regions in the *S*. *singularior* and *Drosophila hsp70* genes showed that these regions in both genomes diverged dramatically, effectively sharing only HSEs and TATA boxes, which also exhibited characteristic differences.

It has been demonstrated that most of *Drosophila hsp* genes are characterized by the presence of several GAGA elements located near the TATA box spaced 20–60 bp from each other [27, 28]. The induction of *hsp70* in different *Drosophila* species relies on two proteins, HSF and GAGA-binding factor (GAF), which resides on *hsp70* promoters prior to heat shock [5]. The binding of GAF maintains the promoter region in a nucleosome-free conformation and establishes paused Pol II and a promoter architecture conducive to binding by HSF [24]. The existence of an open chromatin conformation for the *hsp70* promoter under non-induced conditions is also necessary for the rapid access of general transcription factors (GTFs) and the assembly of the preinitiation complex after HS [6,37,38].

Because the absence of canonical GAGA elements is characteristic of all *S*. *singularior hsp70* promoters, we decided to examine whether these motifs are responsible for the observed dramatic differences in the promoter strength in *Drosophila* cells. Our experiments demonstrated that the absence of functional GAGA elements in the *S*. *singularior hsp70* regulatory region is apparently a key characteristic that determines differences between *D*. *melanogaster* and *S*. *singularior hsp70* promoter strength at least *in vitro* in S2 cells. Our experiments with different numbers and positions of experimentally inserted GAGA elements revealed the need of several closely spaced GAGA sequences in promoters for effective transcription in *Drosophila* cells. The accumulated data corroborated the results of other studies that demonstrated that the presence of multiple clustered GAGA elements is required for efficient GAF binding [24,39]. Moreover, it has been demonstrated that GAF binds more efficiently to GAGA sequences located close to a nucleosomal edge or within the linker DNA [40,41].

It is necessary to mention that in our previous study [21], we detected several GAG sequences scattered in *S*. *singularior* regulatory regions, but the binding experiments reported here exploring *Drosophila* GAF showed that *S*. *singularior* promoters are not capable of binding GAF and apparently the detected single GAG motifs are not functional at least in *D*. *melanogaster* cells. Thus, when we inserted canonical GAGA elements into otherwise intact *S*. *singularior hsp70* promoters, they exhibited heat-induced activity comparable to that of *D*. *melanogaster* endogenous promoters and even significantly exceeded (several times) the activity of *D*. *melanogaster* wild type promoters under steady conditions. It is of note that the *S*. *singularior hsp83* promoter is highly homologous to that of *D*. *melanogaster* and exhibits similar strength both under steady conditions and after HS in *D*. *melanogaster* cells (S2). This result may be explained by the different roles played by the *Hsp70* and *Hsp83* genes in response to stress and under normal physiological conditions in Diptera species cells [42].

The development of strains transformed with constructs containing promoters of different structures enabled us to compare our *in vitro* results (luciferase assay in S2 cells) with *in vivo* data.

In our transformation experiments, we made use of a *D*. *melanogaster* strain in which all six copies belonging to the *hsp70* family (87A and 87C loci) have been deleted [31], which enabled us to monitor the expression of transgenes containing orthologous copies of the *S*. *singularior hsp70* gene.

After HS, we detected only trace levels of *hsp70* transcription in all strains transformed with constructs under the control of the *S*. *singularior* native promoter, and puffs were not formed at the sites of such construct insertions. Surprisingly, constructs containing the *S*. *singularior* promoter with an experimentally inserted optimal number of canonical GAGA elements also exhibited low constitutive transcription comparable with that of *D*. *melanogaster* and modest induction by HS, which was observed only in the larvae. However, even in these series of experiments, the puffs were not formed, which is in contrast with the strains transformed with constructs under the control of the wild-type *D*. *melanogaster* promoter where, as expected, there was strong induction of *hsp70* mRNA, the synthesis of the corresponding protein and well-developed puffs after HS were always observed. The differences in the activity in the I(S-GAGA) promoters in larvae and adult flies (Fig. 4 and Fig. 5) correlates with the previously demonstrated dichotomy in constitutive Hsp70 levels observed between the larvae and adults in Stratiomyidae species [20].

Notably, species-specific differences in HS promoter strength have been described by other groups [9–16]. Thus, the heat-inducible activity of the promoter region of the *D*. *melanogaster hsp70* gene, which contained motifs for HSF and GAF binding assayed in the medfly *Ceratitis capitata* germ-line carrying the *lacZ* reporter, was found to be several fold lower than the activity of the orthologous region of the medfly *hsp70* gene with the same *lacZ* reporter [17]. However, together, our studies clearly demonstrate that *in vitro* data on the promoter strength may not correspond with *in vivo* observations because in the latter case, the heat shock response involves complex changes in the chromatin and histone structure including the formation of active chromatin, the preinitiation complex and redistribution of many regulatory proteins [7,34,43]. Because our intensive computer search (MEGA and MatInspector) failed to detect any potential protein-binding sites in the promoters of the compared species that differentiate their regulatory regions, it is possible that long-range interactions involving some distant regulatory sequences (insulators-enhancers) may determine the observed differences in *in vivo* expression in *hsp7*0 genes, particularly under steady conditions.

Multiple studies have shown that GAGA elements are not the only motifs that may play an important role in *hsp70* gene induction by HS in flies. Thus, the *hsp70* promoters in the fly *Liriomyza sativae* harbor AT-rich sequence elements (ATRS) that are absent in the congeneric species. Deletions of the ATRS from promoters augmented both the constitutive and heat-shock-inducible expression of the luciferase reporter [44]. Intriguingly, the authors detected sites for the binding of the *Zeste* transcription factor within these ATRSs, which may play the role of GAF in this Diptera species, somehow enhancing the strength of the *hsp70* promoter [45]. Along these lines, although we described a correlation between the presence of functional GAGA elements in the otherwise highly diverged *hsp70* promoters and their strength *in vivo* and *in vitro*, the situation is not simple. Thus, it is well known that several *Drosophila* heat-induced genes, including *hsp68*, belonging to the Hsp70 family do not contain functional GAGA elements but are strongly induced by HS and form large puffs after temperature elevation [6].

Therefore, one may conclude that the pre-heat shock nucleosome-free conformation of heat shock gene chromatin, which leads to the extremely fast induction of these loci after HS and other stressful conditions, may be established by the interaction of different transcription factors. Surprisingly, these factors apparently vary not only between species of the same order (e.g., *Drosophila* vs. *Stratiomys*) but within the genomes of the same species (*Drosophila hsp70* genes vs. *hsp68* gene), which makes *hsp* promoters a unique model for the study of the evolutionary significance of regulatory mutations in response to environmental challenges. Thus, a complex species-specific architecture of *hsp70* promoters may be established by multiple interacting factors depending on species evolution occurring under specific environmental conditions [17]. It is well known that stable Pol II pausing at the inducible *hsp70* promoter controlled by several factors is characteristic of non-heat-shocked cells in mammals and Diptera species. However, in cells with a high constitutive level of Hsp70, such pausing may be incomplete, and it is a challenge to determine regulatory factors underlying this phenomenon.

Notably, the behavior of general transcription initiation factors (GTFs), including TFIID, which comprise the TATA box-binding protein (TBP) and TBP-associated factors (TAFs), is most likely more conserved when different species and taxa are compared [7].

The detected variability in mammalian and insect HS promoter regulation characteristics demonstrates that the heat shock regulation system is not universal for distant species, and different trends in the evolution of individual HS genes take place and may in principle depend on environmental conditions.

However, available data do not allow for speculation of whether the differences observed in the *Hsp70* promoters of thermally contrasting species somehow resemble adaptive changes evolved under high temperature condition environments. However, it becomes clear that high Hsp70 content observed in many thermo-resistant species without HS at least in part depends on the DNA sequence of their promoters and yet unidentified regulatory factors (work in progress).

## Materials and Methods

### Sequence analysis

The programs NCBI BLAST and Vector NTI were used to align investigated sequences. The detection of regulatory motifs within the *hsp70* and *hsp83* promoters was performed with the MEGA 4.0 [46] and MatInspector (GENOMATIX Software) programs. Relevant sequence information concerning all investigated *hsp70* genes including regulatory regions is accessible in GenBank. The GenBank accession numbers for *hsp70S1*, *hsp70S2* and *hsp70S3* are HQ184404 and HQ184405 for *hsp70S4* and *hsp70S5* [21]. The GenBank accession numbers for the two *S*. *singularior hsp83* genes are JN627861 and JN627862, respectively [22]. The *D*. *melanogaster hsp70Aa* and *hsp83* genes can be found in FlyBase in the context of the *D*. *melanogaster* genome. A cluster of camel *HSPA1* genes has been deposited in GenBank under the accession number JF837187 [19], while human *HSPA1* genes from the orthologous region are referenced in GenBank as BA000025.

### Construction of luciferase reporter plasmids

Plasmids with fused promoter and luciferase reporter genes were constructed using a promoter/enhancer-free pGL4.10[luc2] basic vector (Promega). DNA fragments containing different portions of the *hsp70* and *hsp83* promoters were amplified using PCR with specific primers containing different restriction sites at the 5’-ends (S1 Table and S2 Table), and they were ligated upstream of the luciferase ORF into the pGL4.10 vector. DNA fragments previously isolated from λ phage genomic libraries of *C*. *dromedarius* and *S*. *singularior* [19,21] were used as a template for cloning the *hsp* promoters of these species. For PCR amplification of the *D*. *melanogaster* and human promoters, genomic DNA isolated from *D*. *melanogaster* flies and human HDF cell culture was used. Different overlapping primer pairs were used to obtain constructs with insertions of GAGA elements or substitutions within the TATA box of the *S*. *singularior hsp70S3* gene upstream of the ATG in the camel *HSPA1A* gene (S1 Table). The promoters of the *hsp83* genes were amplified by PCR from *D*. *melanogaster* genomic DNA and recombinant λ phage containing a part of *S*. *singularior hsp83* cluster [22]. The resulting plasmids were transformed into competent *E*. *coli* DH5a strains for amplification. Endotoxin-free plasmid DNA was isolated using the Plasmid Plus Midi Kit (Qiagen). All constructs were confirmed by restriction analysis and DNA sequencing. Full schemes of all obtained constructs with indicated boundaries and directions are provided in S3 Fig., S4 Fig., and S5 Fig.

### Cell lines, heat shock treatment and luciferase assay

For measurement of mammalian *HSP70* promoter activity, reporter constructs were expressed in the human cell lines HEK293 and HDF [47,48] cultured in DMEM/F12 medium supplemented with 10% fetal bovine serum for luciferase reporter assay experiments. The activity of the Diptera *hsp70* and *hsp83* promoters was determined in *D*. *melanogaster* Schneider-2 cells (S2) in Schneider’s Insect Medium (Sigma) supplemented with 10% fetal bovine serum (HyClone). The TransFast (Promega) reagent was routinely used for the transfection experiments according to the manufacturer’s instructions. Both a firefly luciferase reporter gene construct (200 ng) and a pRL-SV40 *Renilla* luciferase construct (20 ng for normalization of transfection efficiency) were cotransfected into each well of 24-well plates. After transfection, human cells were incubated at 37°C for 48 h, placed in an incubator at either 37°C (control) or 43.5°C (heat shock) for 30 min and then incubated for 5 h at 37°C. S2 cells were incubated at 25°C and heat shocked at 37°C for 30 min with subsequent recovery for 5 h at 25°C. Luciferase activity was measured using the Dual-Luciferase Reporter Assay System (Promega) with The Reporter Microplate luminometer (Turner Designs). The ratio of Firefly to *Renilla* luciferase activities was taken as the activity of reporter construct. The activity of each reporter construct was measured at least in five independent experiments. The significance of differences in the reporter gene expression was estimated using Fisher’s test.

### *Drosophila* fly strains and heat shock

For *P* element-mediated transformation experiments, we used the *D*. *melanogaster* (*w[1118]; Df(3R)Hsp70A*, *Df(3R)Hsp70B*) strain (strain 8841 in Bloomington *Drosophila* Stock Centre) in which all inducible *hsp70* genes from the 87A and 87B loci were deleted [31]. Protein extracts for EMSA were obtained from the *D*. *melanogaster w[1118]* strain. Flies were reared at 25°C on standard sugar-yeast-agar medium containing propionic acid as a mold inhibitor. For heat shock, larvae or adult flies were transferred into preheated 50 ml plastic conical tubes (40 individuals/vial) for temperature stress in a water bath (37°C during 30 min) without anesthesia. After heat treatment, flies or larvae were frozen in liquid nitrogen immediately or after a recovery period and were used for RNA or protein extraction for 5’-RACE, Q-RT-PCR and EMSA experiments. For *in situ* hybridization, larvae were grown at 18°C on standard medium for 2 days before dissection, and for heat treatment, approximately 20 larvae were placed into 50 ml Falcon tubes with a moistened piece of filter paper and incubated in a water bath (37°C for 20 min).

### The development of plasmids for transformation and construction of transgenic flies

The I(S) construct includes an *Eco*RV-*Xba*I fragment from the *S*. *singularior hsp70S3* gene (4663 bp), containing an ORF (1917 bp), a 5’-UTR and 3’-UTR (230 and 247 bp correspondingly), a 5’-flanking region (2117 bp) and a 3’-flanking (152 bp) sequence. This fragment was obtained from λ phage containing a portion of the *S*. *singularior hsp70* cluster [21], cloned into the pBluescriptSK+ vector and then subcloned into pCaSpeR5 transformation vector. To obtain an I(S-GAGA) construct with three GAGA element insertions, step-by-step PCR reactions with overlapping GAGA-containing primers (see S1 Table) were performed. Resulting DNA fragments were inserted into the I(S) construct with replacement of the wild-type promoter.

In construct II(M), we substituted the *S*. *singularior hsp70* promoter and 5’-UTR with a 1557 bp PCR fragment containing the promoter and 5’-UTR region of the *D*. *melanogaster hsp70Aa* gene (primer sequences are given in S3 Table). Next, we excised a *Pst*I-*Xba*I fragment containing the *hsp70S3* ORF with a 3’-UTR and 3’-flanking sequence from the I(S) construct, ligated this fragment with the *Bam*HI-*Pst*I excision of the *D*. *melanogaster* PCR fragment into pBluescriptSK+, and finally subcloned the resulting sequence into pCaSpeR5. All constructs were confirmed by DNA sequencing.

### Expression and purification of the recombinant *D*. *melanogaster* GAF protein

The histidine-tagged GAF fusion protein (His-GAF) contained amino acids 5–519 of D. melanogaster GAF fused in frame with six histidine residues from the pET-28a vector (Novagen, USA). The construct was kindly provided by Dr. E.M. Baricheva of the Institute of Cytology and Genetics, Russia, Novosibirsk. The *Escherichia coli* BL21 strain was used for protein expression. His-GAGA protein purification is described in [25].

### Preparation of protein extracts

Protein extracts were obtained from control and heat-shocked adult *D*. *melanogaster* flies, S2 cells and HEK293 cells. Up to 10^8^ S2 or HEK293 cells were collected after heat shock or from control conditions (see above) and frozen in liquid nitrogen and further processed as described in [49]. *D*. *melanogaster* flies were taken from control conditions or stressed at 37°C and processed as in [25].

### Electrophoretic mobility shift assay (EMSA)

Protein extracts from S2 and HEK293 cells were used in EMSA experiments with fragments from *hsp70* promoters from Diptera and mammalian species (camel, human), respectively. EMSA with protein extracts from *D*. *melanogaster* flies and from S2 cells (10 μg of total protein) was performed as in [25] with ^32^P-labeled oligonucleotides probes of *hsp70S3*, *hsp70S4* and *hsp70Aa* sequences (see S3 Table for primer sequences). *Drosophila* anti-HSF rabbit polyclonal antibodies (kind gift of Prof. C. Wu, NIH) were used in supershift experiments. HEK293 protein extracts (10 μg) were shifted with ^32^P-labelled PCR fragments from human or camel *HSPA1L* promoters (see S3 Table for primers sequences). For EMSA with recombinant *D*. *melanogaster* GAF, oligonucleotides corresponding to the *S*. *singularior hsp70S3* and *hsp70S4* and a *D*. *melanogaster hsp70Aa* promoter were amplified by PCR with specific primers (S3 Table) in the presence of α^32^P-dATP. The binding reaction mixture contained 1 μg of recombinant GAF, 5 ng of (^32^P)-labeled DNA probe, and 10 ng of poly (dI-dC) in the band shift buffer [25]. The reaction mixtures were separated in a 4.5% AA/Tris/borate/EDTA gel. Gels were dried immediately after electrophoresis and exposed to X-ray film.

### *P* Element-Mediated Transformation of *D*. *melanogaster*

Plasmid DNA of the constructs I(S), I(S-GAGA) and II(M) was purified using the QIAprep Spin Miniprep Kit (Qiagen) and used for embryo injection as previously described [50]. Transposase activity was provided by the helper plasmid Turbo Δ2–3, containing a stable genomic source of *P* element transposase from *D*. *melanogaster* [51]; the recipient embryos were from *D*. *melanogaster* strain 8841, which lacks all *hsp70* genes [31]. Adults emerging from the injected embryos were back crossed with flies from the 8841 strain of the opposite sex, and the eye color of their progeny was examined. Transformed strains homozygous for the transgene were established using full sibling mating. The localization of homozygous transgene copies and ability of construction to puff generation was performed using *in situ* hybridization with a *white* biotinylated probe as described in [52]. Transgene intactness was checked by PCR with primers specific for the *P* element and *hsp70* flanking sequences, and transcription start sites of all three constructs in transformed strains were determined by 5’-RACE as described in [21]. For primer sequences, see S4 Table.

### Quantitative Real-time PCR

Total RNA was isolated from larvae or adult flies using TRIzol (Invitrogen). cDNA was prepared from 1 μg of total RNA treated with DNase 1 (Ambion, USA) using random decamer primers and MMLV reverse transcriptase (Evrogen). Experiments were performed using an ABI PRISM7500 Sequence Detection System (Applied Biosystems, USA). Each reaction was performed with a minimum of 3 biological replicates and performed using 2.5 X SYBR Green 1 PCR Master Mix in the presence of ROX reference dye (Syntol) in accordance with the manufacturer’s protocol. Reaction efficiency was evaluated and was at least 95%. Quantification was normalized to the ubiquitously expressed *rpl32* (ribosomal protein L32) and *ef1a* (elongation factor 1α) genes, and the ddCt method was used to calculate relative expression levels. Primer sequences are provided in S3 Table. The significance of differences detected in the experiments was estimated using Fisher’s test.

## Supporting Information

S1 Fig General arrangement of the constructs under control of human and camel promoters *HSPA1L* (left orientation indicated by bent arrows) and *HSPA1A* (right orientation indicated by bent arrows). The relative positions of major regulatory elements and boundaries of promoter regions with respect to the transcription start sites are indicated. The strength of promoters in relation to the controls under non-heat shock conditions (37°C) and after HS (43.5°C) are given in arbitrary units (*renilla* luciferase luminescence). Signal intensity driven by constructs (pFF/pRL) is the ratio of the intensity of luminescence of firefly (pFF) and *renilla* (pRL) luciferase.
(JPG)

S2 Fig General arrangement and expression levels of the constructs under control of human and camel promoters of *HSPA1B* gene. (JPG)

S3 Fig General arrangement of constructs comprising various promoter regions of *D. melanogaster and S. singularior hsp70* and *hsp83* genes. The efficiency of promoters under normal physiological temperature (25°C) and after heat shock (37°C) is represented in arbitrary units resembling luminescence. Major components of promoters (HSEs, TATA-boxes and GAGA motifs) are represented by boxes of different color. Thick green arrows beneath the lines indicate the sites of canonical GAGA elements insertions. Transcription starts are marked by bent arrows.
(JPG)

S4 Fig The alignment of sequences including intergenic region between *HSPA1L* and *HSPA1A* genes and 5`-UTR of camel and human *HSPA1L*. The transcription direction of both genes is indicated by arrows. Transcription start points are marked by arrows. TATA-boxes and HSEs are given in red while 112 bps primate-specific sequence is colored blue.
(JPG)

S5 Fig The alignment of *S. singularior* (*hsp70S3*) and *D. melanogaster* (*hsp70Aa*) *hsp70* genes. Transcription start is marked by asterisk and a bold letter, TATA boxes, HSEs and the first ATG codon are underlined. GAGA sites within *D*. *melanogaster hsp70Aa* promoter are colored red.
(JPG)

S1 Table Primers, used for obtaining of *H. sapiens* and *C. dromedarius hsp70* constructs. (DOC)

S2 Table Primers, used for obtaining of *S. singularior* and *D. melanogaster hsp70* and *hsp83* constructs. (DOC)

S3 Table A. Primers used for *D. melanogaster hsp70Aa* regulatory region amplification for II(M) construct; B. Primers for obtaining of fragments used in EMSA with HEK293 extracts; C. Primers for obtaining of fragments used in EMSA with recombinant GAF; D. Overlapping oligonucleotides used for EMSA experiments with HSF-HSE binding; E. Primers for Q-RT-PCR analysis. (DOC)

S4 Table Primers used for the 5’-RACE analysis. (DOC)
