# Supplementary figures and images for: Activity of Heat Shock Genes’ Promoters in Thermally Contrasting Animal Species
Source: PLoS One. 2015 Feb 20;10(2):e0115536. doi: 10.1371/journal.pone.0115536 (PMC4336284; doi:10.1371/journal.pone.0115536)

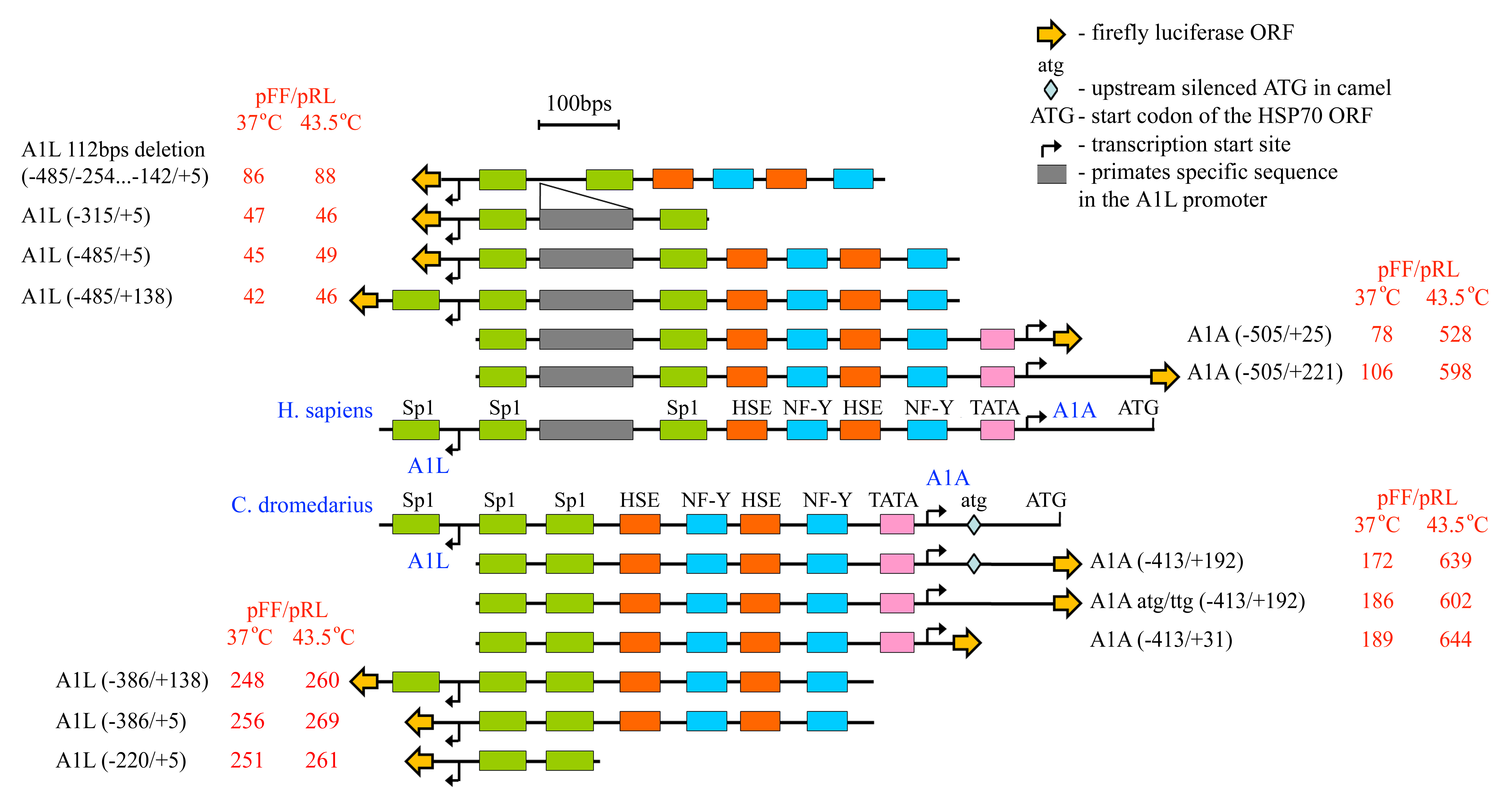

Supplement: S1 Fig — The relative positions of major regulatory elements and boundaries of promoter regions with respect to the transcription start sites are indicated. The strength of promoters in relation to the controls under non-heat shock conditions (37°C) and after HS (43.5°C) are given in arbitrary units (renilla luciferase luminescence). Signal intensity driven by constructs (pFF/pRL) is the ratio of the intensity of luminescence of firefly (pFF) and renilla (pRL) luciferase. (JPG) [file pone.0115536.s001.jpg]

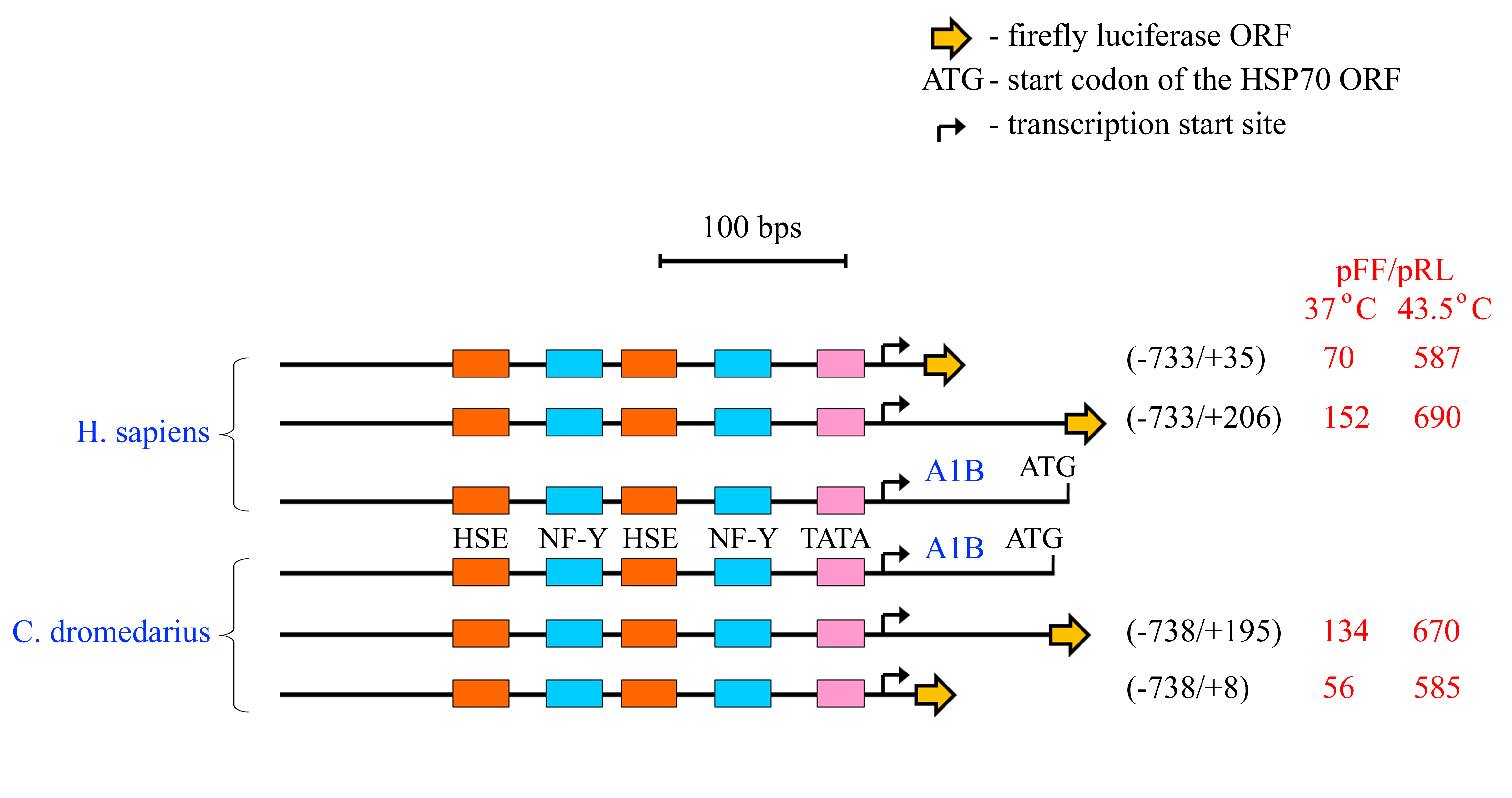

Supplement: S2 Fig — (JPG) [file pone.0115536.s002.jpg]

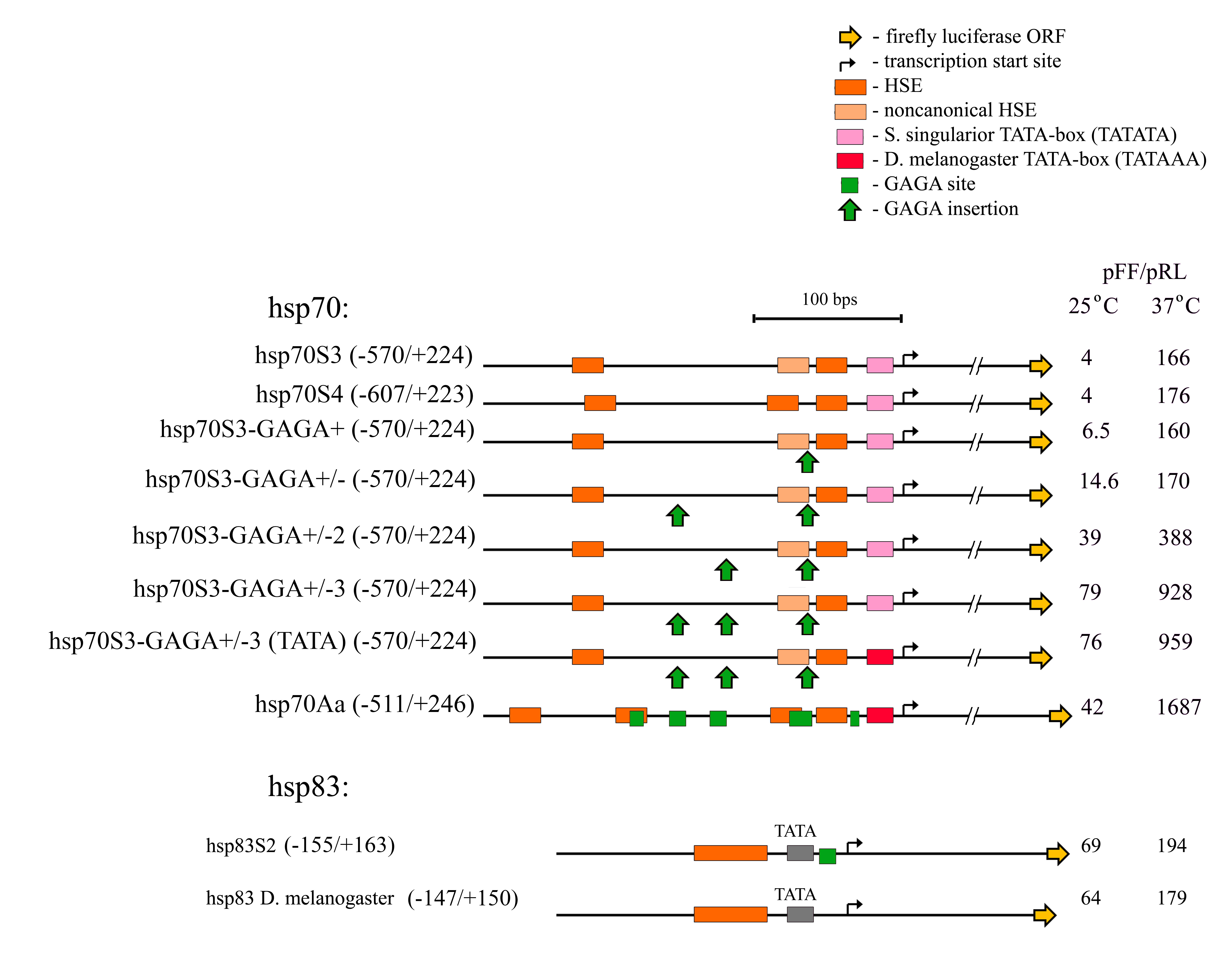

Supplement: S3 Fig — The efficiency of promoters under normal physiological temperature (25°C) and after heat shock (37°C) is represented in arbitrary units resembling luminescence. Major components of promoters (HSEs, TATA-boxes and GAGA motifs) are represented by boxes of different color. Thick green arrows beneath the lines indicate the sites of canonical GAGA elements insertions. Transcription starts are marked by bent arrows. (JPG) [file pone.0115536.s003.jpg]

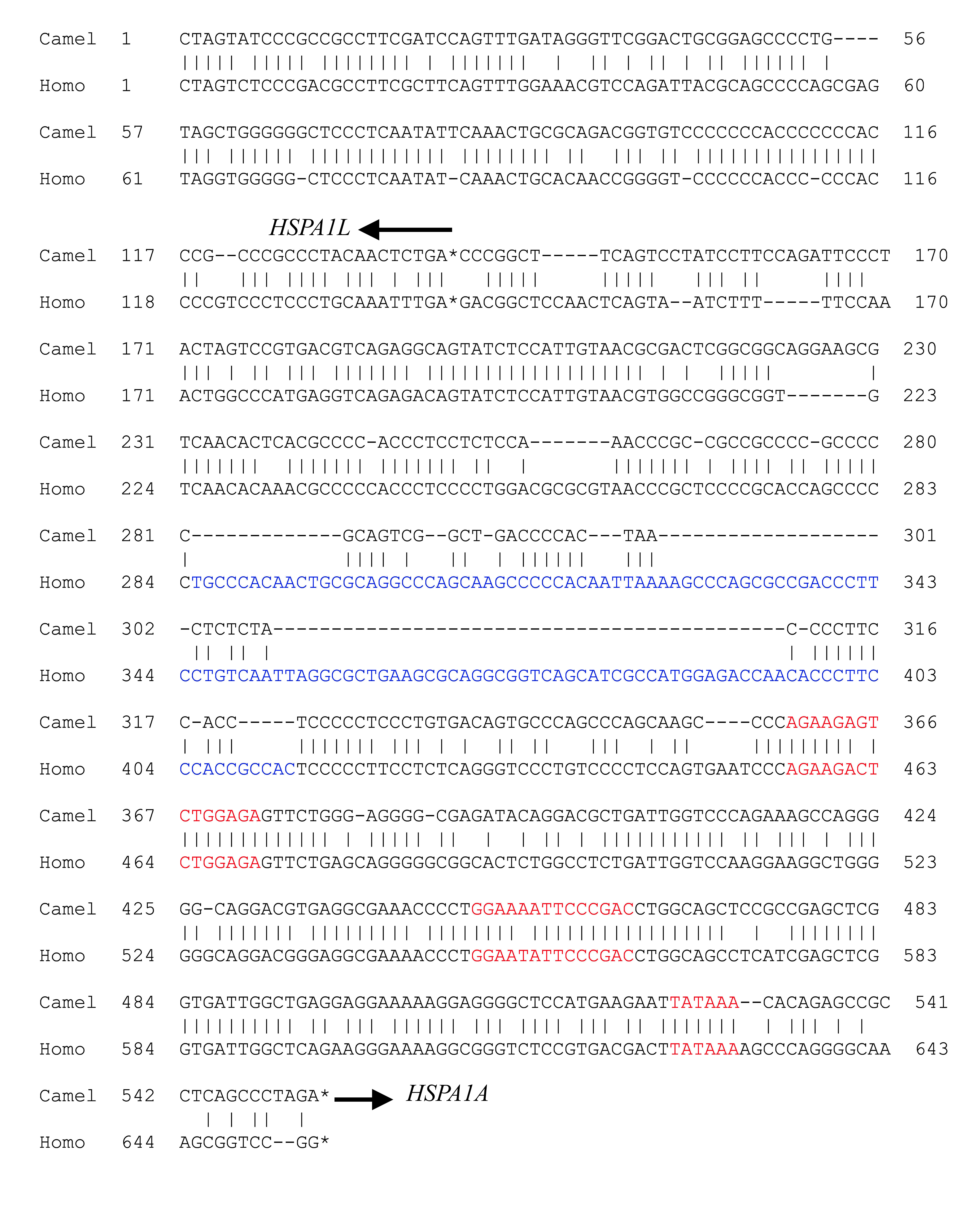

Supplement: S4 Fig — The transcription direction of both genes is indicated by arrows. Transcription start points are marked by arrows. TATA-boxes and HSEs are given in red while 112 bps primate-specific sequence is colored blue. (JPG) [file pone.0115536.s004.jpg]

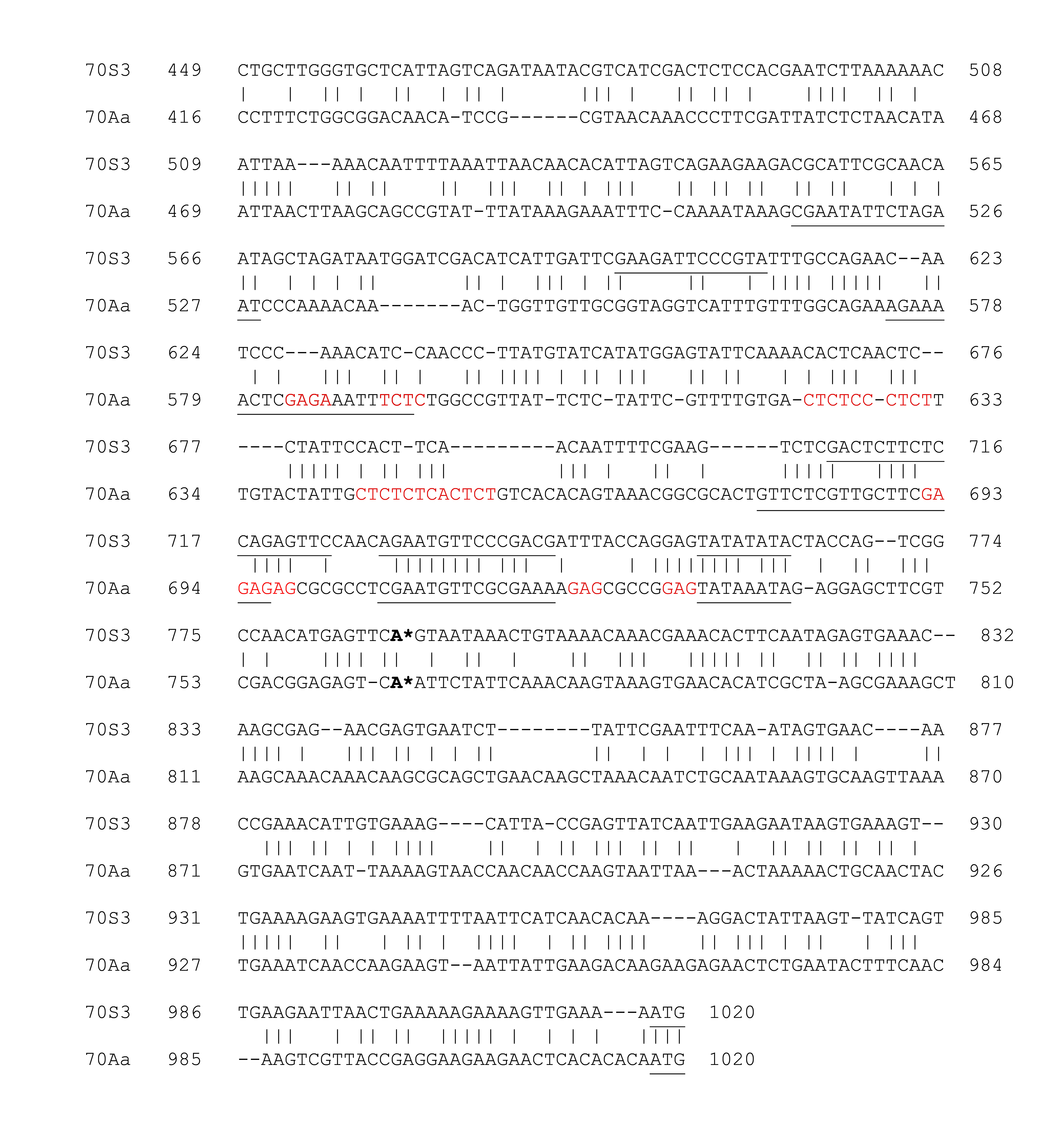

Supplement: S5 Fig — Transcription start is marked by asterisk and a bold letter, TATA boxes, HSEs and the first ATG codon are underlined. GAGA sites within D. melanogaster hsp70Aa promoter are colored red. (JPG) [file pone.0115536.s005.jpg]
