# Supplementary material for: Activity of Heat Shock Genes’ Promoters in Thermally Contrasting Animal Species
Source: PLoS One. 2015 Feb 20;10(2):e0115536. doi: 10.1371/journal.pone.0115536 (PMC4336284; doi:10.1371/journal.pone.0115536)
Supplement: S1 Table — (DOC) [file pone.0115536.s006.doc]

Table S1. Primers, used for obtaining of *H. sapiens* and *C. dromedarius* *hsp70* constructs

| N | Species | Primers for PCR amplification and mutagenesis | Gene | Start/end relatively to transcription initiation point | Cloning sites |
| --- | --- | --- | --- | --- | --- |
| 1 | *Н. sapiens* | F: AAGGTACCGACGGCTCCAACTCAGTAATCT  R: AAAAGCTTCTCAGGCTAGCCGTTATCCG | *HSPА1А* | -505/+25 | KpnI/HindIII |
| 2 | *Н. sapiens* | F: AAGGTACCGACGGCTCCAACTCAGTAATCT  R: AAAAGCTTGGTTCCCTGCTCTCTGTCGG | *HSPA1A* | -505/+221 | KpnI/HindIII |
| 3 | *Н. sapiens* | F: ATAAGCTTATTTGAGACGGCTCCAACTCAG  R: AAGGTACCAGTCGTCACGGAGACCCGCCT | *HSPA1L* | -485/+5 | KpnI/HindIII |
| 4 | *Н. sapiens* | F: TGAAGCTTTAGTCTCCCGACGCCTTCGC  R: AAGGTACCAGTCGTCACGGAGACCCGCCT | *HSPA1L* | -485/+138 | KpnI/HindIII |
| 5 | *Н. sapiens* | F1: ATAAGCTTATTTGAGACGGCTCCAACTCAG  F2: TTCCTGCAGGACACCCTTCCCACCGCCACTC  R1: AAGGTACCAGTCGTCACGGAGACCCGCCT  R2: TTCCTGCAGGGGGCTGGTGCGGGGAGCGG | *HSPA1L* | -485/-254…-142/+5  112 bps deletion | KpnI/SbfI  SbfI/HindIII |
| 6 | *Н. sapiens* | F: ATAAGCTTATTTGAGACGGCTCCAACTCAG  R: TTGGTACCGGGATTCACTGGAGGGGACAGGG | *HSPA1L* | -315/+5 | KpnI/HindIII |
| 7 | *Н. sapiens* | F: GCGGTACCCACTGCTCCCATTACC  R: AAAAGCTTACCCTCGCAGCAGCTCCTCA | *HSPA1B* | -733/+35 | KpnI/HindIII |
| 8 | *Н. sapiens* | F: GCGGTACCCACTGCTCCCATTACC  R: ATAAGCTTTGCTCTGTGGGCTCCGCTCT | *HSPA1B* | -733/+206 | KpnI/HindIII |
| 9 | *C. dromedarius* | F: TTGGTACCCGGCTTCAGTCCTAT  R: AAAAGCTTCGCAACTCCCGTCTCAG | *HSPА1А* | -413/+31 | KpnI/HindIII |
| 10 | *C. dromedarius* | F: TTGGTACCCGGCTTCAGTCCTAT  R: ATAAGCTTTTCTCCGCCCCGCAA | *HSPА1А* | -413/+192 | KpnI/HindIII |
| 11 | *C. dromedarius* | F1: TTGGTACCCGGCTTCAGTCCTAT  F2: TTCTGCAGACCCACTGCTTCCGAGGA  R1: ATAAGCTTTTCTCCGCCCCGCAA  R2: GGTCTGCAGAAGCTGTCCTCACGGACTACA**a**AACGCAAC | *HSPА1А* | -413/+192  atg-TTG | KpnI/PstI  PstI/HindIII |
| 12 | *C. dromedarius* | F: TTAAGCTTCTCTGACCCGGCTTCAGTCC  R: TTGGTACCTTCTTCATGGAGCCCCTCCT | *HSPA1L* | -386/+5 | KpnI/HindIII |
| 13 | *C. dromedarius* | F: TGAAGCTTCCTAGTATCCCGCCGCCTTCG  R: TTGGTACCTTCTTCATGGAGCCCCTCCT | *HSPA1L* | -386/+138 | KpnI/HindIII |
| 14 | *C. dromedarius* | F: TTAAGCTTCTCTGACCCGGCTTCAGTCC  R: TTGGTACCGGGGCTTGCTGGGCTGGGCAC | *HSPA1L* | -220/+5 | KpnI/HindIII |
| 15 | *C. dromedarius* | F: TTGGTACCTGATTGCTTCTCTTTTGA  R: ATAAGCTTGCGGTTTCTCTGAGG | *HSPA1B* | -738/+8 | KpnI/HindIII |
| 16 | *C. dromedarius* | F: TTGGTACCTGATTGCTTCTCTTTTGA  R: ATAAGCTTTTCTCCGCCCCGCAATGA | *HSPA1B* | -738/+195 | KpnI/HindIII |
