# Supplementary material for: Activity of Heat Shock Genes’ Promoters in Thermally Contrasting Animal Species
Source: PLoS One. 2015 Feb 20;10(2):e0115536. doi: 10.1371/journal.pone.0115536 (PMC4336284; doi:10.1371/journal.pone.0115536)
Supplement: S2 Table — (DOC) [file pone.0115536.s007.doc]

Table S2. Primers, used for obtaining of *S. singularior* and *D. melanogaster* *hsp70* and *hsp83* constructs

| N | Construct | Primers for PCR amplification and mutagenesis | Gene | Start/end relatively to transcription initiation point | Cloning sites |
| --- | --- | --- | --- | --- | --- |
| 1 | *Hsp70Aa* | F: ATGGTACCCAGTTGACAACAACAGTCTTGA  R: AAAAGCTTGTGAGTTCTTCTTCCTCGGTA | *Hsp70Aa* | -511/+246 | KpnI/HindIII |
| 2 | *Hsp70S3* | F: TTGGTACCAGTGCTTGATGCATATTGTTCCA  R: GGAAGCTTCTTTTTCTTTTTCAGTTAATTCTTCAA | *Hsp70S3* | -570/+224 | KpnI/HindIII |
| 3 | *Hsp70S3-GAGA+* | F1: TTGGTACCAGTGCTTGATGCATATTGTTCCA  R1: GGAAGCTTCTTTTTCTTTTTCAGTTAATTCTTCAA  F2: TCCC**TCGAGAGAGC**GTTCCAACAGAATGTTCCCGACGA  R2: TAACTCGAGTGGAGAAGAGTCGAGACTTCGA | *Hsp70S3* | -570/+224 | KpnI/XhoI  XhoI/HindIII |
| 4 | *Hsp70S3-GAGA+/-* | F1: TTGGTACCAGTGCTTGATGCATATTGTTCCA  R1: GGAAGCTTCTTTTTCTTTTTCAGTTAATTCTTCAA  F2: ATCATATGGAGTATTCAAAACACTCA  R2: TCCATA**TGAGAGAGC**TACATAAGGGTTGGATGTTTGGG | *Hsp70S3* | -570/+224 | KpnI/NdeI  NdeI/HindIII |
| 5 | *Hsp70S3-GAGA+/-2* | F1: TTGGTACCAGTGCTTGATGCATATTGTTCCA  R1: GAAGTGGAATAC**AGAG**T**GAGAGAG**CGTTTTGAATACTCCATATG  F2: CATATGGAGTATTCAAAACG**CTCTCTC**A**CTCT**GTATTCCACTTC  R2: GGAAGCTTCTTTTTCTTTTTCAGTTAATTCTTCAA | *Hsp70S3* | -570/+224 | KpnI/HindIII |
| 6 | *Hsp70S3-GAGA+/-3* | F1: TTGGTACCAGTGCTTGATGCATATTGTTCCA  R1: GAAGTGGAATAC**AGAG**T**GAGAGAG**CGTTTTGAATACTCCATATG  F2: CATATGGAGTATTCAAAACG**CTCTCTC**A**CTCT**GTATTCCACTTC  R2: GGAAGCTTCTTTTTCTTTTTCAGTTAATTCTTCAA | *Hsp70S3* | -570/+224 | KpnI/HindIII |
| 7 | *Hsp70S3-GAGA+/-3 (TATA)* | F1: TTGGTACCAGTGCTTGATGCATATTGTTCCA  R1: TGGCCGACTGGTAGTAT**t**TATACTCCTGGTAAATCGTCGGGA  F2: ATGTTCCCGACGATTTACCAGGAGTATA**a**ATACTACCAGTCGGCC  R2: GGAAGCTTCTTTTTCTTTTTCAGTTAATTCTTCAA | *Hsp70S3* | -570/+224 | KpnI/HindIII |
| 8 | *Hsp70S4* | F: TTGGTACCTTCACAATAGAAATGGTATC  R: TTAGATCTCTTTTCTTTGAGAGTTAATTCTTCAA | *Hsp70S4* | -607/+223 | KpnI/BglII |
| 9 | *Hsp83 D. melanogaster* | F:ATGGTACCAGCTTGCACCACCAAGTC  R: TTAAGCTTGTATGTATGTTTTTCGTTCTA | *Hsp83* | -147/+150 | KpnI/HindIII |
| 10 | *Hsp83S2* | F:ATGGTACCTTCTTACATGCAATGCTATCGG  R: AAAAGCTTGACTGGATTCTTCAAAAACTTCT | *Hsp83S2* | -155/+163 | KpnI/HindIII |
