# Supplementary material for: Activity of Heat Shock Genes’ Promoters in Thermally Contrasting Animal Species
Source: PLoS One. 2015 Feb 20;10(2):e0115536. doi: 10.1371/journal.pone.0115536 (PMC4336284; doi:10.1371/journal.pone.0115536)
Supplement: S3 Table — (DOC) [file pone.0115536.s008.doc]

Table S3.

1. Primers used for *D. melanogaster hsp70Aa* regulatory region amplification for II(M) construct

| Sequence | Direction |
| --- | --- |
| TCCGGATCCTTAAATTGTATCCT | Direct |
| TACTGCAGGCATTGTGTGTGAGTTCT | Reverse |

1. Primers for obtaining of fragments used in EMSA with HEK293 extracts

| Sequence | Direction |
| --- | --- |
| AGGGTGGGGGCGTTTGTGTTGA  ACAAACGCCCCCACCCTCCCCTGC | Homo1 Forward  Homo2 Reverse |
| TTGTGGGGGCTTGCTGGGC  GCCCAGCAAGCCCCCACAA | Homo -112 Forward  Homo -112 Reverse |
| GGGTGGGGCGTGAGTGTTGA  TTAAGCTTCTCTGACCCGGCTTCAGTCC | Camel Forward  Camel Reverse |

1. Primers for obtaining of fragments used in EMSA with recombinant GAF

| Sequence | Gene |
| --- | --- |
| CTGTTCTCGTTGCTTCGAGA  TTGACTCTCCGTCGACGA | *hsp70Aa* |
| CGAAGTCTCGACTCTTCTCCAG  GAACTCATGTTGGCCGACTG | *hsp70S3* and *hsp70S3-GAGA+/-* |
| AAACGTTCGAAGCTTCTCGA  TGAACTCATGTTGGCCGACTG | *hsp70S4* |

1. Overlapping nucleotides used for EMSA experiments with HSF-HSE binding

| Sequence | Gene |
| --- | --- |
| atccgagcgcgcctcgaatgttctagaa  ccttttctagaacattcgaggcgcgctc | *hsp70Aa* |
| ttttcgaagtctcgactcttctccagagttccaacagaatgttcccg  taaatcgtcgggaacattctgttggaactctggagaagagtcgagac | *hsp70S3* |
| cgttcgaagcttctcgatacatctacagagttccaacagaatgttcccg  taaatcgtcgggaacattctgttggaactctgtagatgtatcgag | *hsp70S4* |

1. Primers for Q-RT-PCR analysis

| Sequence | Gene |
| --- | --- |
| CGATTCGAGGAACTGTGTGCG  CACCATATGCCACCGCCTCAT | *hsp70S3* |
| GCGTGGGTTTGTGATCAGTT  GATCTTCTCCTTGCCCATCC | *ef1a* |
| ATGCTAAGCTGTCGCACAAAT  GTTCGATCCGTAACCGATGT | *rpl32* |
