# Supplementary material for: Activity of Heat Shock Genes’ Promoters in Thermally Contrasting Animal Species
Source: PLoS One. 2015 Feb 20;10(2):e0115536. doi: 10.1371/journal.pone.0115536 (PMC4336284; doi:10.1371/journal.pone.0115536)
Supplement: S4 Table — (DOC) [file pone.0115536.s009.doc]

Table S4.

Primer used for the 5’-RACE analysis

| TGTTCTTCGGGTTCATAGCCAC | *S. singularior hsp70S3 and S4*, 5’-RACE, the first |
| --- | --- |
| CAATTCCTCAAGTCCTCCTG | *S. singularior hsp70S3 and S4*, 5’-RACE, the second |
| AATCTGTGAAAGCCACGTAG | *D. melanogaster hsp70Aa*, 5’-RACE, the first |
| GGTTGCCCTGGTCGTTGGCG | *D. melanogaster hsp70Aa*, 5’-RACE, the second |
